# Evaluation of limited-sampling strategies to calculate AUC_(0–24)_ and the role of *CYP3A5* in Chilean pediatric kidney recipients using extended-release tacrolimus

**DOI:** 10.3389/fphar.2023.1044050

**Published:** 2023-03-14

**Authors:** Carla Galvez, Pía Boza, Mariluz González, Catalina Hormazabal, Marlene Encina, Manuel Azócar, Luis E. Castañeda, Angélica Rojo, María Luisa Ceballos, Paola Krall

**Affiliations:** ^1^ Unidad de Nefrología, Hospital Luis Calvo Mackenna, Santiago de Chile, Chile; ^2^ Laboratorio Clínico, Hospital Luis Calvo Mackenna, Santiago de Chile, Chile; ^3^ Servicio de Farmacia Clínica, Hospital Luis Calvo Mackenna, Santiago de Chile, Chile; ^4^ Programa de Genética Humana, Instituto de Ciencias Biomédicas, Facultad de Medicina, Universidad de Chile, Santiago de Chile, Chile; ^5^ Departamento de Pediatría y Cirugía Infantil Oriente, Facultad de Medicina, Universidad de Chile, Santiago de Chile, Chile; ^6^ Instituto de Medicina, Facultad de Medicina, Universidad Austral de Chile, Valdivia, Chile

**Keywords:** pediatric kidney transplant, tacrolimus, limited sampling strategies, area under a curve (AUC), CYP3A5

## Abstract

**Background:** Kidney transplantation (KTx) requires immunosuppressive drugs such as Tacrolimus (TAC) which is mainly metabolized by CYP3A5. TAC is routinely monitored by trough levels (C_0_) although it has not shown to be a reliable marker. The area-under-curve (AUC) is a more realistic measure of drug exposure, but sampling is challenging in pediatric patients. Limited-sampling strategies (LSS) have been developed to estimate AUC. Herein, we aimed to determine AUC_(0–24)_ and *CYP3A5* genotype in Chilean pediatric kidney recipients using extended-release TAC, to evaluate different LSS-AUC_(0–24)_ formulas and dose requirements.

**Patients and methods:** We analyzed pediatric kidney recipients using different extended-release TAC brands to determine their trapezoidal AUC_(0–24)_ and *CYP3A5* genotypes (SNP rs776746). Daily TAC dose (TAC-D mg/kg) and AUC_(0–24)_ normalized by dose were compared between CYP3A5 expressors (*1/*1 and *1/*3) and non-expressors (*3/*3). We evaluated the single and combined time-points to identify the best LSS-AUC_(0–24)_ model. We compared the performance of this model with two pediatric LSS-AUC_(0–24)_ equations for clinical validation.

**Results:** Fifty-one pharmacokinetic profiles were obtained from kidney recipients (age 13.1 ± 2.9 years). When normalizing AUC_(0–24)_ by TAC-D significant differences were found between CYP3A5 expressors and non-expressors (1701.9 vs. 2718.1 ng*h/mL/mg/kg, *p* < 0.05). C_0_ had a poor fit with AUC_(0–24)_ (*r* ^2^ = 0.5011). The model which included C_0_, C_1_ and C_4_, showed the best performance to predict LSS-AUC_(0–24)_ (*r* ^2^ = 0.8765) and yielded the lowest precision error (7.1% ± 6.4%) with the lowest fraction (9.8%) of deviated AUC_(0–24)_, in comparison to other LSS equations.

**Conclusion:** Estimation of LSS-AUC_(0–24)_ with 3 time-points is an advisable and clinically useful option for pediatric kidney recipients using extended-release TAC to provide better guidance of decisions if toxicity or drug inefficacy is suspected. The different *CYP3A5* genotypes associated with variable dose requirements reinforce considering genotyping before KTx. Further multi-centric studies with admixed cohorts are needed to determine the short- and long-term clinical benefits.

## Introduction

Kidney transplantation (KTx) is the therapy of choice for pediatric patients who have reached end-stage kidney disease (ESKD) offering lower mortality and a better quality of life (MacDonald et al., 2004). Kidney recipients require immunosuppressive therapy to prevent graft rejection. The calcineurin inhibitor Tacrolimus (TAC) is the immunosuppressor most widely used given its optimal tolerability and improved graft survival (Dugast et al., 2016). TAC is available as immediate- and extended-release capsules administered twice or once a day, respectively, with no apparent differences in terms of providing equivalent exposure (McCormack, 2014). Extended-release TAC is associated with improvements in medication adherence, potentially achieving better outcomes (Guirado et al., 2011; Kuypers et al., 2013). Depending on the institutional guidelines and experience, the post-transplantation period can be started with extended-release capsules or alternatively with immediate-release capsules switching later to extended-release capsules, displaying similar clinical outcomes during the first year (Ho et al., 2019).

TAC is characterized by a narrow therapeutic range and high variability among patients. For this reason, plasmatic TAC levels undergo constant monitoring throughout the post-transplantation period to avoid supra- or sub-therapeutic levels, given that both scenarios increase the risks of adverse events (Staatz and Tett, 2004). The evidence has shown that the estimation of the area-under-curve in 24 h, AUC_(0–24)_, represents a realistic marker of drug exposure when using extended-release TAC formulations, although it requires sampling at multiple time-points that might be inconvenient in specific cases. To facilitate the clinical routine, TAC is habitually monitored by trough levels (C_0_) to guide clinical decisions and avoid side effects, but it has not shown to be a reliable marker with a suboptimal correlation with AUC_(0–24)_ (Wong et al., 2000; Pisitkun et al., 2002). Although AUC_(0–24)_ has proven to be a more accurate marker, repeated sampling can be difficult in pediatric or outpatient populations (Scholten et al., 2005). To face this challenge, limited-sampling strategies (LSS) have been developed and proposed as an optimal choice to estimate AUC_(0–24)_ in pediatric recipients using TAC, but these equations need to be assayed in different geographic or ethnic populations.

TAC pharmacokinetics is influenced by many factors, including the activity of the liver enzyme CYP3A5, whose gene contains the best-studied single nucleotide polymorphism, SNP rs776746 (c.6986A>G), described as the most relevant in TAC metabolism (Oetting et al., 2018). The A>G nucleotide substitution (CYP3A5*3 allele) causes a splicing defect that determines that CYP3A5*3/*3 patients, also known as non-expressors, have null enzyme activity and consequently low drug requirements to achieve target TAC levels. In contrast, those patients carrying at least one wild-type CYP3A5*1 allele (genotypes CYP3A5*1/*3 and CYP3A5*1/*1), also known as expressors, usually require TAC doses 1.5–2.0 fold higher than the standard doses (Kuehl et al., 2001; Busi and Cresteil, 2005; Birdwell et al., 2015). The distribution of these *CYP3A5* alleles has shown to differ considerably between geographic populations and the presence of the three *CYP3A5* genotypes has been described in Chile previously, each one of them associated with significant differences in TAC dose requirements in a short period after KTx (Zong et al., 2017; Krall et al., 2021).

There are limited studies focused on the development of LSS-AUC_(0–24)_ equations in pediatric kidney recipients using different brands of extended-release TAC and carrying different *CYP3A5* genotypes. Herein, we aimed to evaluate the utility of single and combined time-points to predict an accurate AUC_(0–24)_ with LSS and to explore the role of *CYP3A5*. Additionally, we compared the performance of the best LSS model with two pediatric LSS-AUC_(0–24)_ equations described previously for clinical validation (Almeida-Paulo et al., 2014).

## Patients and methods

### Patient data and ethical aspects

Pediatric patients that had undergone KTx between 2006 and August 2021 at the referral transplant center Hospital Luis Calvo Mackenna (Santiago, Chile) and were receiving stable doses of extended-release TAC, were invited to participate in this observational, prospective and longitudinal study. In our center, around 80% of the transplantations were performed with deceased donors during this period of years. We studied 51 recipients who received TAC combined with mycophenolic acid as immunosuppressive therapy with or without steroids, as described previously by our group (Delucchi et al., 2007). All the patients were considered Chileans if born (natives) or living (inhabitants) in Chile and whose parents had historical, ethnic or cultural connections with the country.

The study was conducted according to the declaration of Helsinki with the approval from the Ethic Committee of the Universidad de Chile and from the Director of the Hospital Luis Calvo Mackenna. Parental or legal tutors written informed consent was requested in all cases and patients aged between 12 and 17 years old were asked for assent as their affirmative agreement to participate in this study. Patients with combined liver-kidney transplantation, evidence of graft dysfunction at the time of the study, or parents that denied participation were excluded.

### Therapeutic TAC monitoring

Therapeutic drug monitoring of TAC was applied regularly to all recipients and dose adjustment was performed whenever necessary to achieve the target C_0_ defined in our center (10–15 ng/mL during the first 3 months and 5–7 ng/mL after the third-month post-KTx). The analysis of AUC_(0–24)_ was conducted on recipients mainly during outpatient visits but also during hospitalization, with stable immunosuppression based on TAC therapy. At the time of the patient’s schedule for AUC_(0–24)_, the daily TAC dose normalized by weight (TAC-D mg/kg) was registered to compare later CYP3A5 expressors and non-expressors. In our center, target AUC_(0–24)_ values range from 170 to 250 ng*h/mL during the maintenance phase (Tanzi et al., 2016). Extended-release TAC brands were not the same in all patients, but are considered bioequivalent according to local regulatory agencies and were administered as follows: Prograf XL^®^ n = 38, Tacni XR^®^ n = 7 and Cidimus XL^®^ n = 3. Three patients were receiving Prograf XL^®^ combined with Tacni XR^®^ or Cidiums XL^®^ capsules and they were excluded from the analyses comparing observed trapezoidal AUC_(0–24)_ between innovator (Prograf XL^®^) and bioequivalent users (Tacni XR^®^ or Cidimus XL^®^) or considered mixed when analyzing LSS-predicted vs. observed AUC_(0–24)_.

### Trapezoidal AUC_0–24_

In all patients, 2 mL blood samples were taken in EDTA tubes at 0 h (pre-dose; C_0_) and after TAC oral dose intake at the following times: 1 (C_1_), 2 (C_2_), 4 (C_4_), 12 (C_12_), and 24 (C_24_) hours. Then, plasma TAC concentrations were measured by performing an immunoassay using the Abbott Architect i1000 instrument (Abbott Laboratories). To estimate the AUC_0-24_ for each patient, we integrated the plasma TAC concentrations against time using the *Bolstad2* package for R (Curran, 2022). This procedure gives the same AUC_0-24_ values as the gold standard trapezoidal method. The C_max_ (highest TAC concentration) and T_max_ (timepoint with the highest TAC concentration) were also registered.

### Role of CYP3A5 in AUC_(0–24)_ and TAC dose

DNA was extracted from whole peripheral blood samples using the QIAmp DNA Blood Mini Kit (QIAGEN) following the manufacturer’s instructions. The *CYP3A5* genotype (SNP rs776746) was determined using the TaqMan assay^®^ or the PCR-RFLP technique with specific primers and the restriction enzyme *SspI*, according to the described protocol (Rong et al., 2010). Each assay included controls confirmed previously by Sanger sequencing for the three genotypes: homozygous reference allele (CYP3A5*1/*1), heterozygous (CYP3A5*1/*3) and homozygous variant allele (CYP3A5*3/*3). Negative controls with an equal volume of nuclease-free pure water were included. We classified patients’ *CYP3A5* genotypes according to the reported effect on CYP3A5 expression: CYP3A5 expressors (genotypes CYP3A5*1/*1 and *1/*3) and CYP3A5 non-expressors (genotype CYP3A5*3/*3).

Analysis of variance (ANOVA) was performed to compare AUC_(0–24)_ values among CYP3A5 genotypes, CYP3A5 expression groups, and TAC brand. We also compared TAC-D and AUC_(0–24)_ normalized by TAC-D between CYP3A5 expression groups by performing ANOVA. For these analyses, we checked the normality of ANOVAs’ residuals and tested the homogeneity of variance using the Fligner-Killen test. s. Differences among groups were considered statistically significant if the *p*-value was lower than 0.05.

### LSS-AUC_0-24_ pharmacokinetic and statistical analysis

To develop an LSS for pediatric patients, we performed multiple regression analyses using C_0_, C_1_, C_2,_ and C_4_ as predictor variables and AUC_0-24_ as the response variable. We avoided the use of C_12_ and C_24_ to develop an LSS where samples were suitable for the outpatient pediatric population. Regression models including all combinations of C_0_, C_1_, C_2_ and C_4_ were obtained using the *olsrr* package for R (Hebbali, 2020). This procedure generated 15 different regression models from which we selected those with an adjusted *r* ^2^ value higher than 0.799 (Ting et al., 2006). The regression models that matched this criterion were retained and their performances were compared using the *performance* package for R that includes the estimations of the Akaike information criterion (AIC) and Bayesian information criterion (BIC) weights (Lüdecke et al., 2021). AIC and BIC weights represent the probability that a given model is the best among a set of retained LSS models.

We also evaluated the precision of the retained LSS models calculating the percentage of the absolute prediction error (APE) that was considered for validation of the LSS equations (Op den Buijsch et al., 2007). APE was calculated as: APE(%) = 100*|(LSS-AUC_(0–24)_-trapezoidal AUC_(0–24)_)|/trapezoidal AUC_(0–24)_. Median APE (MAPE) was calculated and compared between the different LSS equations.

### Clinical validation for LSS-AUC_0-24_ equations

We compared the precision of LSS-AUC_(0–24)_ equation with the best performance identified in this study with two global equations developed previously in pediatric patients from Spain that included together liver and kidney transplants using immediate- or extended-release TAC capsules (Almeida-Paulo et al., 2014):

$$LSS-\left(C_{0}-C_{1}-C_{4}\right)-{AUC}_{\left(0-24\right)}=39.179+8.772*C_{0}+2.297*C_{1}+7.926*C_{4}$$ (1)

$$LSS-\left(C_{0}-C_{2}-C_{4}\right)-{AUC}_{\left(0-24\right)}=46.062+9.129*C_{0}+2.768*C_{2}+6.450*C_{4}$$ (2)

MAPE was calculated and compared between the different LSS-AUC_(0–24)_ equations. Given the high pharmacokinetic variability, APE values of less than 15% were considered clinically acceptable. In addition, Bland–Altman plots for the three LSS-AUC_(0–24)_ equations were used to analyze the agreement between LSS-predicted and observed AUC_(0–24)_ expressed as the difference between methods (method A- method B) vs. methods A-B average and to compare the limits of agreement with 95% CI around the average difference.

## Results

### Basic and clinical patients characteristics

Altogether 51 pediatric recipients were included that had undergone KTx at a single transplant center at 7.8 ± 4.0 years, being the structural anomalies the leading cause of ESKD (Table 1). At the time of the patient’s recruitment, the age was 13.1 ± 2.9 with a mean time since KTx of 5.3 years (range: min 6 months-max 15.2 years). The daily TAC-D requirements were 0.108 ± 0.049 (range: min 0.039- max 0.228) mg/kg with a predominant (80%) use of Prograf XL^®^ as a single TAC brand or mixed with other brands.

**TABLE 1:** Basic demographic, clinical and genetic information of Chilean kidney recipients.

Characteristics	
Male/female	31/20
*Cause of ESKD*	
Structural anomaly	32 (62.7%)
Glomerulopathy	8 (15.7%)
Cystic disease	5 (9.8%)
Monogenic cause (confirmed)	1 (2.0%)
Other	3 (5.9%)
Undetermined or unknown	2 (3.9%)
Age at transplantation (mean ± SD)	7.8 ± 4.1 years
Age at study recruitment (mean ± SD) [min-max]	13.1 ± 2.9 [7–18] years
*TAC brand*	
Prograf XL^®^	38 (74.5%)
Tacni XR^®^	7 (13.7%)
Cidimus XL^®^	3 (5.9%)
Prograf XL^®^ with Tacni XR^®^ or Cidiums XL^®^	3 (5.9%)
TAC dose/weight (mean ± SD)	0.108 ± 0.049 mg/kg
*CYP3A5 genotype (n)*	
*1/*1	2
*1/*3	14
*3/*3	32
Missing	3
Trapezoidal AUC_(0–24)_ (mean ± SD)	225 ± 59 ng*h/mL

ESKD, end-stage kidney disease; SD, standard deviation; TAC, Tacrolmus; AUC_(0–24)_, area under the curve in 24 h.

The *CYP3A5* genotypes were distributed as follows: 4.2% presented the CYP3A5*1/*1 genotype, 29.2% the heterozygous CYP3A5*1/*3 genotype and 66.6% the CYP3A5*3/*3 genotype (Table 1). Altogether, 33.4% were CYP3A5 expressors and 66.6% were CYP3A5 non-expressors. The frequency of the CYP3A5*3 variant allele resulted in 81.3%. Genotype and allele frequencies were not significantly different than expected if the population was in Hardy-Weinberg equilibrium (Goodness fit test: χ2 = 0.09, *p*-value = 0.77).

**FIGURE 1:**
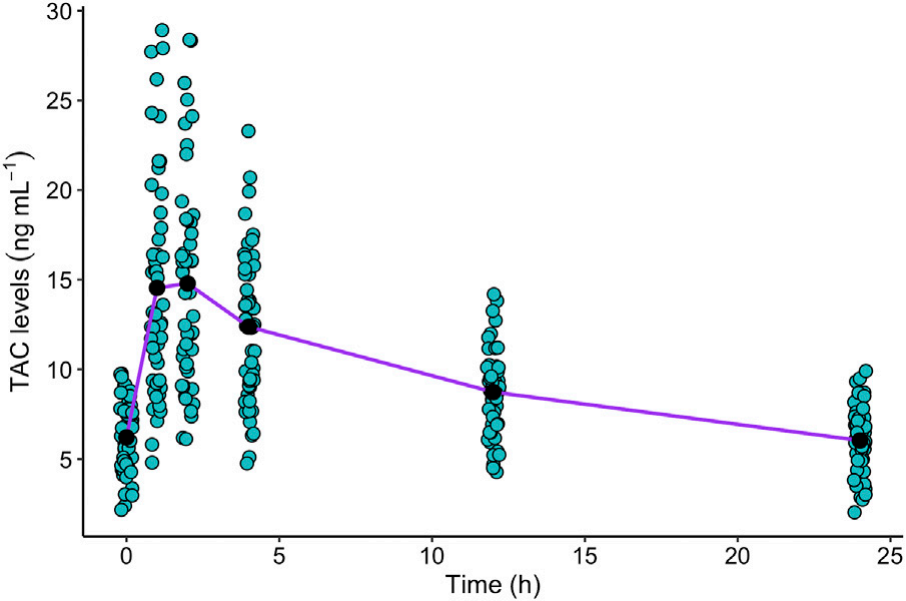
Plasmatic TAC levels in 24 h monitoring, were obtained in children and adolescent kidney recipients (n = 51) at C_0_, C_1_, C_2_, C_4_, C_12_ and C_24_ timepoints. We represented with dots each TAC level to be able to visualize the dispersion of the data. The black dots represent the mean TAC concentration at each time-point. The purple line represents the mean TAC concentration-time curve.

Trapezoidal AUC_(0–24)_ did not differ significantly among *CYP3A5* genotypes (ANOVA: F_2,45_ = 2.30, *p*-value = 0.11), CYP3A5 expression groups (ANOVA: F_2,46_ = 0.32, *p*-value = 0.58) or TAC brand (ANOVA: F_2,43_ = 0.34, *p*-value = 0.85). Then, AUC_(0–24)_ values were pooled for the following analyses. The mean AUC_(0–24)_ was 225 ± 52 (range: min 122—max 361.4) ng*h/mL and the curves fit a two-compartmental model (Figure 1). The mean C_0_ level was 6.2 ± 2.0 ng/mL and we identified 36/51 (70.6%) cases out of the therapeutic range defined in the transplant center. The mean C_max_ was 16.7 ± 6.1 (range: min 6.4—max 28.9 ng/mL) and T_max_ occurred mainly at C_1_ (39.2%) and C_2_ (33.3%) time-points.

**FIGURE 2:**
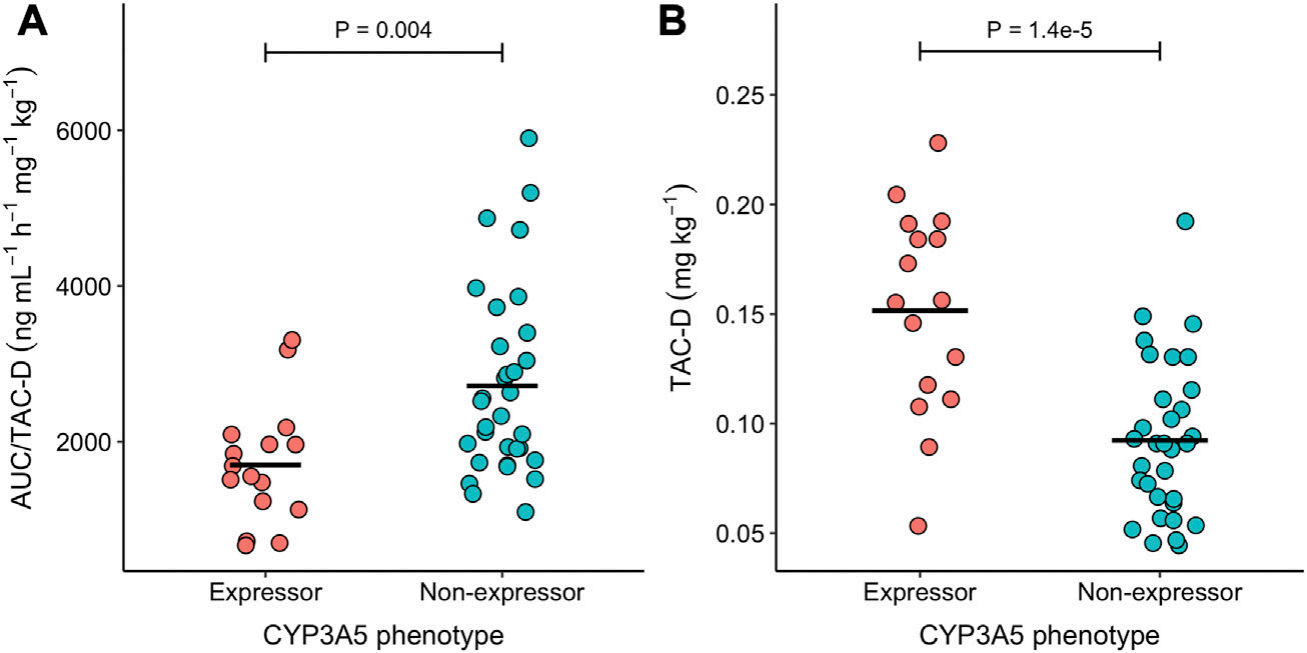
Scatterdot plot of the **(A)** AUC(0–24)/TAC-D and **(B)** TAC-D in the two CYP3A5 expression groups. Horizontal black lines represent the mean of each group. Differences between expression groups were tested using a Mann Whitney U-test. P-value of these tests is indicated above the whiskers.

When normalizing AUC_(0–24)_ by TAC-D significant differences were found between CYP3A5 expressors and non-expressors (1701.9 vs. 2718.1 ng*h/mL/mg/kg; ANOVA: F_1,46_ = 9.39, *p*-value = 0.004; Figure 2A), which can be explained by the significant increase in TAC-D requirements in CYP3A5 expressors in comparison to CYP3A5 non-expressors (0.145 vs. 0.092 mg/kg; ANOVA: F_1,46_ = 23.69, *p*-value = 1.4 × 10^−5^; Figure 2B). These differences in TAC-D requirements were not influenced by the brands when comparing the innovator and the bioequivalent products in the CYP3A5 non-expressors group (ANOVA: F_1,45_ = 0.05, *p*-value = 0.95).

### LSS-AUC_(0–24)_ models analysis

Our LSS generated 15 predictive models for AUC_(0–24)_ values combining C_0_, C_1_, C_2_, and C_4_ values (Table 2). Based on these results, we observed that the single time-points had a lower fit than models with combined time-points. For instance, C_0_ had a poor fit to determine the trapezoidal AUC_(0–24)_ (*r* ^2^ = 0.5011), while C_4_ had a better predictive value (*r* ^2^ = 0.7350). According to the clinical criterion that the adjusted *r* ^2^ of LSS models should be higher than 0.799, we selected 4 LSS models that combined two (model 7), three (models 12 and 13), or four (model 15) time-points (see bold models in Table 2).

**TABLE 2:** Multiple regression analysis of all combinations with C_0_, C_1_, C_2_ and C_4_ with the adjusted *r* ^2^ for each model. This procedure generated 15 different regression models from which we selected those with an adjusted *r* ^2^ value higher than 0.799 highlighted in bold (see Patients and Methods for more details).

Model	Predictor	Adjusted *R* ^2^
1	C_0_	0.5011
2	C_1_	0.3503
3	C_2_	0.4394
4	C_4_	0.7350
5	C_0_ - C_1_	0.6749
6	C_0_—C_2_	0.6478
**7**	**C**_**0**_ **—C**_**4**_	**0.8532**
8	C_1_—C_2_	0.4599
9	C_1_—C_4_	0.7712
10	C_2_—C_4_	0.7853
11	C_0_ - C_1_—C_2_	0.6880
**12**	**C**_**0**_ **- C**_**1**_ **—C**_**4**_	**0.8839**
**13**	**C**_**0**_ **—C**_**2**_ **—C**_**4**_	**0.8726**
14	C_1_—C_2_—C_4_	0.7886
**15**	**C**_**0**_ **—C**_**1**_ **—C**_**2 **_ **—C**_**4**_	**0.8848**

Then, we assessed performance to determine how well models fit the data. Based on r-squared, MAPE, wAIC, and wBIC, we considered the best LSS was model 12 (*r* ^2^ = 0.8765), which included three time-points, C_0_, C_1_ and C_4_ (Table 3; Supplementary Figure S1). Based on the indices of performance, this model showed the highest probability of being the best of the retained models, although it had shown a very similar adjusted *r* ^2^ with model 15 (*r* ^2^ = 0.8748) which included four time-points (Table 3; Supplementary Figure S1). Then, we highlight the importance of including a model selection approach (using wAIC and wBIC) to compare different LSS.

**TABLE 3:** Limited sampling equations with adjusted *r* ^2^ higher than 0.799. MAPE, AIC weight, BIC weight and performance percentage are shown. The LSS with the best performance has been highlighted in bold.

Model	LSS-AUC_(0–24)_ equation	Adjusted *R* ^2^	MAPE	AICw	BICw	Performance (%)
7	AUC = 36.18 + C_0_*11.53 + C_4_*9.50	0.8471	8.3% ± 6.4%	0.004	0.014	0.00
**12**	**AUC = 26.0+C**_**0**_ *11.27+C_**1**_ *1.97+C_**4**_ *8.13	**0.8765**	**7.1% ± 6.4%**	**0.647**	**0.781**	**99.06**
13	AUC = 31.82 + C_0_*10.26 + C_2_*1.73 + C_4_*8.42	0.8644	7.5% ± 6.2%	0.060	0.072	42.31
15	AUC = 26.30 + C_0_*10.95 + C_1_*1.67 + C_2_*0.49 + C_4_*8.04	0.8748	7.1% ± 6.2%	0.288	0.132	74.57

LSS, limited sampling strategy; MAPE, median absolute precision error; AICw, Akaike information criteria weight; BICw, Bayesian information criteria weight.

In addition, we tested the utility of the LSS model 12 for the CYP3A5 expressor and non-expressor phenotypes and concluded that the equation is robust and valid in both groups (Supplementary Figure S2).

### Clinical validation of LSS-AUC_(0–24)_ models

**FIGURE 3:**
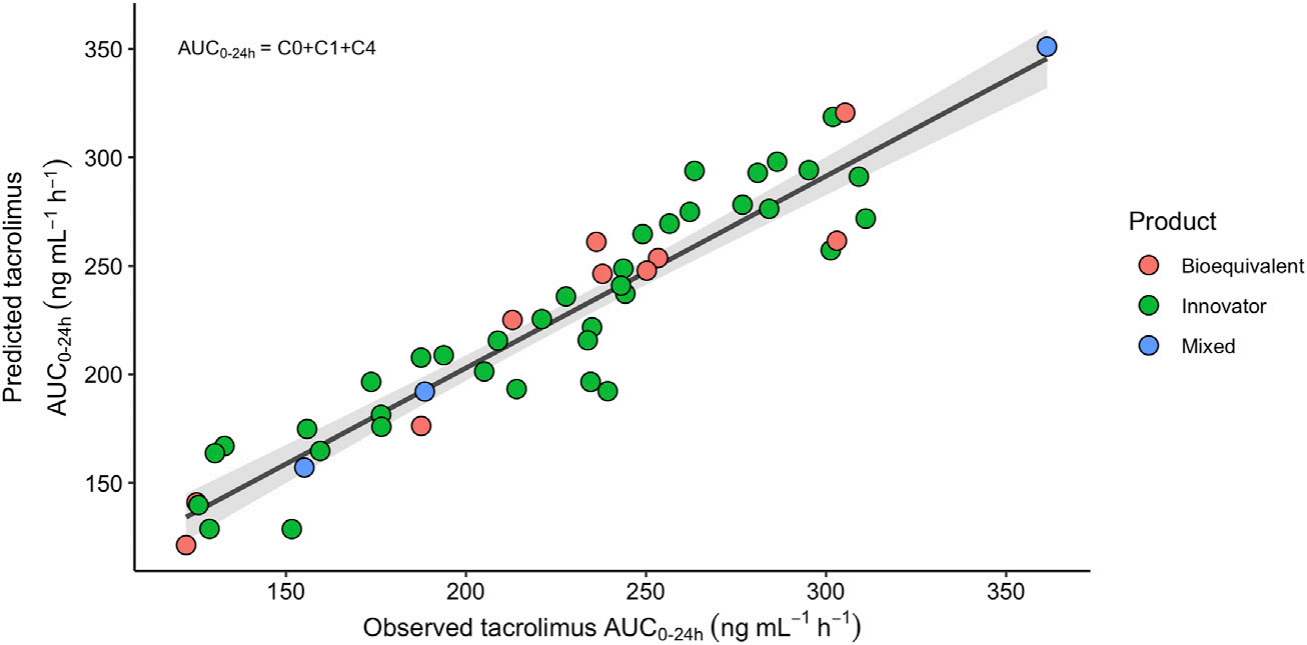
Relationship between the TAC observed trapezoidal and predicted AUC_(0–24)_ according to the equation LSS-AUC_(0–24)_ = 26.0 + 11.27*C_0_+1.97*C_1_+8.13*C_4_, with the innovator (green dots), the bioequivalent (pink dots) or a mixed combination (blue dots) of TAC extended-release products. The solid black line is the regression line and the gray shaded area represents 95% confidence interval for the regression line (*r* ^2^ = 0.8765).

We observed the best performance in model 12 which used extraction points at 0, 1, and 4 h with the following equation: LSS-AUC_(0–24)_ = 26.0 + 11.27*C_0_ + 1.97*C_1_ + 8.13*C_4_. The mean LSS-AUC_(0–24)_ values for this equation was 225 ± 55 (range: min 121- max 351) ng*h/mL. This LSS-AUC_(0–24)_ equation was consistent among different clinical conditions, showing the same trend between patients using the innovator, bioequivalent, or a mixed version of these products, (ANCOVA: F_2,45_ = 0.22, *p*-value = 0.80; Figure 3), different *CYP3A5* genotypes (ANCOVA: F_2,42_ = 0.08, *p*-value = 0.92), different CYP3A5 expression groups (ANCOVA: F_2,44_ = 0.0003, *p*-value = 0.99), or different age groups (<13 yo and >13 yo patients) (ANCOVA: F_2,47_ = 1.68, *p*-value = 0.20).

**FIGURE 4:**
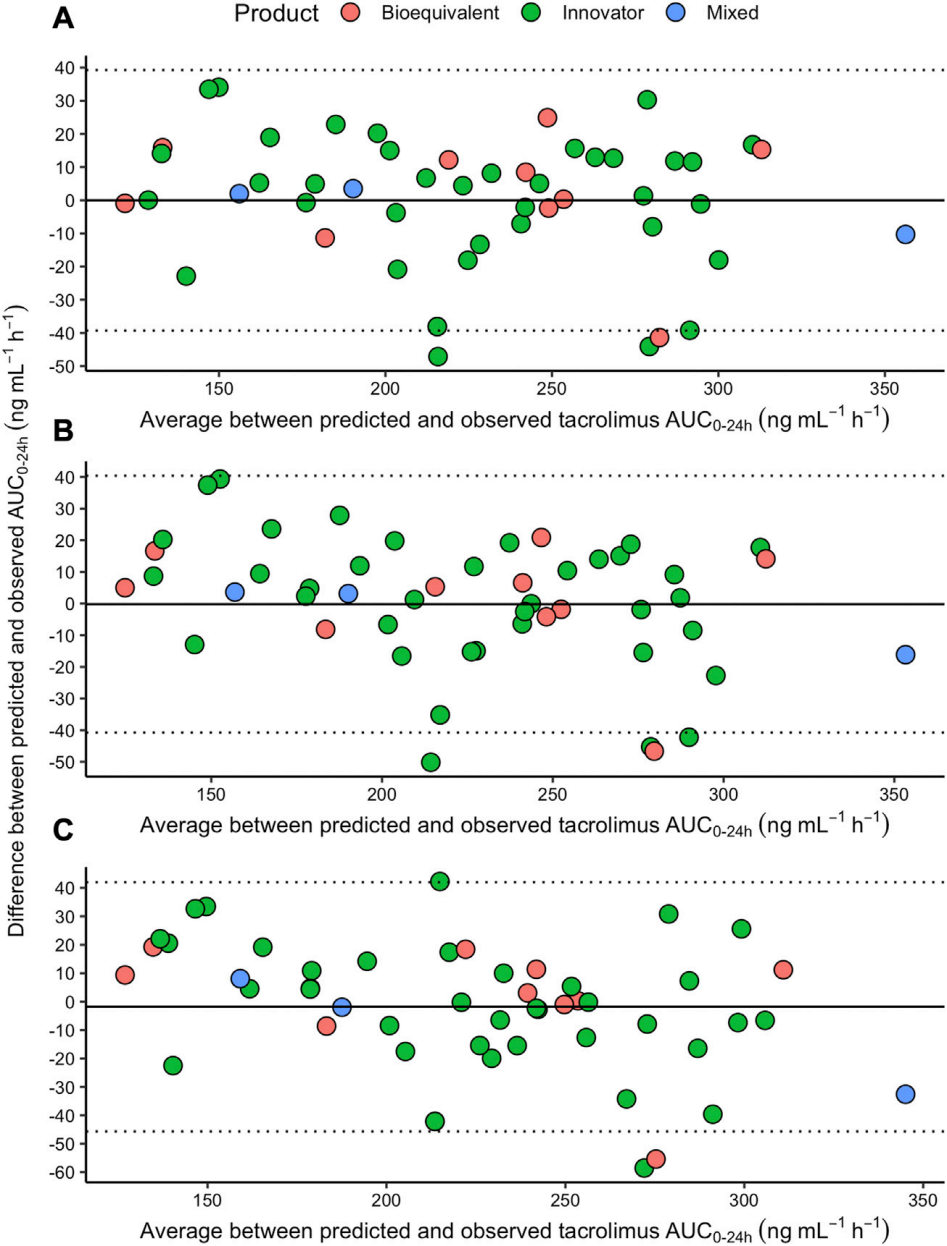
Bland-Altman plot to analyze the agreement between each one of the three LSS-predicted [**(A)** = model 12; **(B)** = model described by Almeida-Paulo using C_0_, C_1_ and C_4_; **(C)** = model described by Almeida-Paulo using C_0_, C_2_ and C_4_] and the observed trapezoidal AUC_(0–24),_ with the innovator (green dots), the bioequivalent (pink dots) or a mixed combination (blue dots) of TAC extended-release products. The dotted lines represent the 95% upper and lower limits. The solid line represents the average of the arithmetic differences between the LSS-predicted and LSS-observed values.

Additionally, we calculated the LSS-AUC_(0–24)_ values using two previously described equations that include three time-points (see Materials and Methods). The mean LSS-AUC_(0–24)_ for the equations described by Almeida-Paulo et al., in 2014 using C_0_-C_1_-C_4_ and C_0_-C_2_-C_4_ values observed in our cohort, yielded 225 ± 52 (range: min 127- max 345) and 223 ± 51 (range: min 129- max 329) ng*h/mL, respectively. Mean values for APE (MAPE) were clinically acceptable for the three LSS-AUC_(0–24)_ equations. However, estimating AUC_(0–24)_ values with the LSS-AUC_(0–24)_ model 12 yielded the lowest MAPE with the lowest fraction (9.8%) of values above 15%. According to the Bland-Altman plots, all three models showed a good concordance with trapezoidal AUC_(0–24)_, with marginally better prediction accuracy in LSS including C_0_-C_1_-C_4_ (Figures 4A–C). Limits of agreement showed a high concordance between the two LSS using C_0_-C_1_-C_4_, and slight differences with the model Almeida-Paulo et al. using C_0_-C_2_-C_4_, but these differences did not reach statistical significance. In addition, the average of the differences between predicted and observed LSS-AUC_(0–24)_ was close to zero in the three plots.

## Discussion

TAC is considered a cornerstone immunosuppressive drug in KTx, but accurate monitoring of drug exposure is mandatory to avoid adverse events. One of the key features is that most pharmacokinetic and/or pharmacogenetic studies in KTx are performed in adults, with less evidence in recipients under 18 years as we present herein. Adolescence and young adulthood are considered critical periods with a high risk of graft loss due to bad adherence (van Arendonk et al., 2013). Furthermore, previous studies in our center demonstrated that non-adherence was the main predictor of graft loss and death, turning immunosuppression monitoring into a primary priority to promote adherence in these patients (Gajardo et al., 2021).

Extended-release TAC capsules were developed later than immediate-release capsules and approved by FDA in 2013. For this reason, multi-center studies and extensive reports have provided strong recommendations regarding immediate-release capsules, while increasing evidence has demonstrated that extended-release capsules are an advisable option as we pretended to explore in this study because they are easier to administer and improve patients’ adherence with the same efficacy (Ho et al., 2013; Meçule et al., 2019).

On average our cohort used daily TAC doses within the same range as other Latin American cohorts in strong association with the *CYP3A5* genotypes (García-Roca et al., 2012; Santoro et al., 2013). The Chilean population, as well as other Latin American countries, originated mainly from the admixture between Native Amerindians and Europeans. This genetic mixture has a clinical impact since it has been documented that CYP3A5*3 is the predominant allele among Europeans reaching 93%–96% frequency. In contrast, Latin American subpopulations with high American ancestry present a 70% frequency of CYP3A5*3 (Gonzalez-Covarrubias et al., 2019). As expected, we found in our cohort an allele frequency between these values, similar to a report with a larger cohort of unrelated Chilean volunteers and estimated Amerindian-Caucasian admixture, facilitating the comparison of different variables between the CYP3A5 subgroups (Roco et al., 2012). The genetic ancestry of our patients was not assayed, but previous studies have shown differences between *CYP3A5* genotype frequencies in Central and Southern Chile, being the last one characterized by a high Mapuche Native Amerindian ancestry (Contreras-Castillo et al., 2021). Our patients were transplanted at a single center in Central Chile but are derived from different cities along Chile which might be associated with an increase in genetic variability. We cannot rule that the genetic ancestries, in combination with the *CYP3A5* genotype, might be contributing to the phenotype variability as has been demonstrated previously (Mohamed et al., 2019).

The AUC_(0–24)_ was not influenced by the *CYP3A5* genotype which can be explained by the long median time since KTx, where the doses were adjusted whenever necessary to assure C_0_ values close to the therapeutic range. However, we observed a 50% increase in TAC requirements in CYP3A5 expressors in comparison to non-expressors, as extensively communicated in retrospective studies and recommended by the Clinical Pharmacogenetics Implementation Consortium (Birdwell et al., 2015). Moreover, transplant patients who receive TAC doses from day 1 adapted to their *CYP3A5* genotype achieve target C_0_ earlier and require fewer dose adjustments than those receiving conventional weight-based doses (Thervet et al., 2010). Despite the availability of strong evidence in favor of *CYP3A5*-adapted dosing, the Chilean Transplant Guideline recommends starting with conventional weight-based TAC dosing without considering the *CYP3A5* genotype but has not been actualized since 2011. If the data of TAC-D are normalized by AUC, similar to the pharmacokinetic parameter known as drug clearance, we observed significantly higher values in CYP3A5 expressors (0.730 L/kg/h) than in non-expressors (0.436 L/kg/h). Although TAC absorption rates are not available, an increased depuration might be assumed in the first group of patients, reinforcing their requirement for higher TAC doses to achieve drug exposure within the target.

Our patients were considered under stable immunosuppression because dose adjustments had been made prior to this study. Although mean C_0_ was within the therapeutic range, more than half of the cases presented values out of range, equally below and above it. On the other hand, mean trapezoidal AUC_(0–24)_ was also within the therapeutic range, but half of the patients were out of range, and twice more frequently above the upper limit. We are unaware if this might cause adverse effects such as toxicity or viral infections more frequently than signs of rejection since clinical outcomes were not evaluated, but this result might be useful in forthcoming studies to evaluate the limits of the therapeutic AUC_(0–24)_ range.

Although C_0_ is routinely used to guide clinical decisions irrespective of the TAC release formulation, we observed that it had a poor correlation with AUC_(0–24)_. Interestingly, C_4_ and C_24_ (data not shown) presented better results, but *r* ^2^ was not acceptable. It should be kept in mind that C_0_ and C_24_ are theoretically the same time-point, but C_24_ values present usually a better correlation with AUC_(0–24)_ and represent a time-point with better supervision of the last capsule intake. It has been suggested that C_24_ might be considered an optimal marker of drug exposure substituting C_0_, but further studies are required to validate this hypothesis and clinical convenience (Rubik et al., 2019).

The development of LSS has a high clinical impact to determine drug exposure without compromising accuracy, patient safety or comfort. We analyzed a homogeneous cohort with the same transplanted organ and TAC release formulation, to select an LSS combining a few extraction time-points in a short period suitable for the routine clinical setting in recipients with different *CYP3A5* genotypes. Previous studies seeking an LSS for adults using extended-release TAC have concluded that the combination of C_10_ or C_12_ with other time-points improves the correlation between LSS-predicted and observed AUC_(0–24)_ but might be not convenient in the pediatric population (El-Nahhas T et al., 2022). We identified only a few studies aiming to develop LSS in pediatric recipients, rarely under extended-release TAC therapy, but most of them conclude that the use of no more than three time-points extracted in maximum 4 h after morning intake, is an ethical-, cost- and a time-effective option to obtain an unbiased and precise prediction of AUC_(0–24)_ (Zhao et al., 2011; Zhao et al., 2013; Almeida-Paulo et al., 2014).

The validation of our LSS equation is desirable to be performed in internal and external cohorts but represents a major challenge in terms of sampling logistics, costs and risks considering the current pandemic scenario, since many patients travel long distances to visit their transplant center and immunocompromised patients have increased risk of illness and death (Kates et al., 2021). However, our study has many advantages to consider our LSS equation acceptable to be used in routine practice. First, the extensive recruitment of patients (n = 51) allowed us to select an LSS model with only a few cases with a trapezoidal AUC_(0–24)_ deviation higher than 15%. Second, after hospital discharge, the AUC_(0–24)_ estimation has limitations in recipients that are not strictly supervised during their daily TAC intakes. Most of our data came from outpatient recipients, conferring this model as a more realistic representation of drug monitoring in underaged patients. Third, our LSS model was compared with ‘global’ LSS equations showing a similar performance, but our model had lower prediction error and AUC_(0–24)_ deviation resulting in a pediatric-friendly LSS with high accuracy and precision in predicting AUC_(0–24)_. Fourth, our LSS model is based on a single equation, which is valid for CYP3A5 both expressor and non-expressor patients.

This study has a variety of limitations. Developing countries face many challenges related to economic constraints and, although the Chilean health system guarantees the immunosuppressive scheme, public drug tendering imposes different TAC brands to treat recipients at transplant centers. Although this might introduce heterogeneity it also contributes to representativeness, and the AUC_(0–24)_ predictions based on our LSS do not seem to be different between the innovator and the bioequivalent products, as observed previously by other authors (Medina-Aymerich et al., 2019). Therefore, the developed limited sampling equation might be used in patients that undergo a switch between brands. A major concern mentioned earlier is therapy adherence which was not considered in our analysis. Many reports refer to a wide range of adherence in recipients less than 18 years with lower success during adolescence, when parents are expected to reduce supervision to gradually acquire a consultant role (Bell, 2022). However, according to our dataset, no significant correlations were found between the age (range 7–18 years) and trapezoidal or LSS-predicted AUC_(0–24)_ values (data not shown).

Clinical trials to define personalized immunosuppressive therapies have been performed mainly on non-Hispanic cohorts of European descendants, while other populations remain underrepresented. Latin Americans are characterized by mixed genetic ancestries and their variable therapeutic response may be caused by multiple genes involved in different biological pathways interacting with epigenetic and environmental factors (Guo et al., 2019; Freitas et al., 2020; Robert et al., 2021). Our patients were Chileans, known to be an admixed population, and the results may be applicable in similar cohorts from Latin America. In our opinion, caution should be taken if our results are considered to be applied to other ethnic or geographic pediatric populations.

In our opinion, the application of LSS-AUC_(0–24)_ is highly recommendable in pediatric kidney recipients who have trough levels that are considered theoretically therapeutic but develop symptoms of drug toxicity or incomplete efficacy. We propose to perform LSS-AUC_(0–24)_ two or three times a year, in complement to routinary trough levels to check if these are similar to those determined as part of previous LSS with AUC_(0–24)_ results considered therapeutic. Taking this decision might avoid excessive sampling during the first years, in accordance with the logistic and financial capacities of each transplant center.

In summary, we have demonstrated that the trough level in pediatric kidney recipients using extended-release TAC is a poor surrogate marker of drug exposure. Based on our results, if a reliable drug exposure needs to be determined we recommend considering LSS-AUC_(0–24)_ with only three time-points, C_0_-C_1_-C_4_, as well as considering *CYP3A5* genotyping to guide TAC dosing after KTx and to identify patients at risk of adverse effects. Further studies focused on short- and long-term clinical outcomes are required, to evaluate the cost-efficiency of LSS-AUC_(0–24)_ and *CYP3A5* genotyping.

## Data Availability

The original contributions presented in the study are publicly available. This data can be found here: https://figshare.com/s/8ca73259623654103720.

## Supplementary material

SUPPLEMENTARY FIGURE S1 Spider chart to visualize five indices of model performance (*r* ^2^, adjusted *r* ^2^, weight BIC (wBIC), weight AIC (wAIC) and MAPE^-1^ (reciprocal value of MAPE) for the four LSS models and allow comparison of indices across models 7 (red line), 12 (green line), 13 (yellow line) and 15 (purple line). The primary descriptors (specific TAC time-points for each AUC model) are shown.

SUPPLEMENTARY FIGURE S2 Relationship between the TAC observed trapezoidal and predicted AUC_(0-24)_ according to the equation LSS-AUC_(0–24)_=26.0+11.27*C_0_+1.97*C_1_+8.13*C_4_, with the expressor (pink dots) or the non-expressor (turquoise dots) CYP3A5 phenotype. The solid black line is the regression line and the gray shaded area represents 95% confidence interval for the regression line (*r* ^2^=0.8765).
