# Supplementary figures and images for: Evaluation of limited-sampling strategies to calculate AUC(0–24) and the role of CYP3A5 in Chilean pediatric kidney recipients using extended-release tacrolimus
Source: Front Pharmacol. 2023 Mar 14;14:1044050. doi: 10.3389/fphar.2023.1044050 (PMC10043346; doi:10.3389/fphar.2023.1044050)

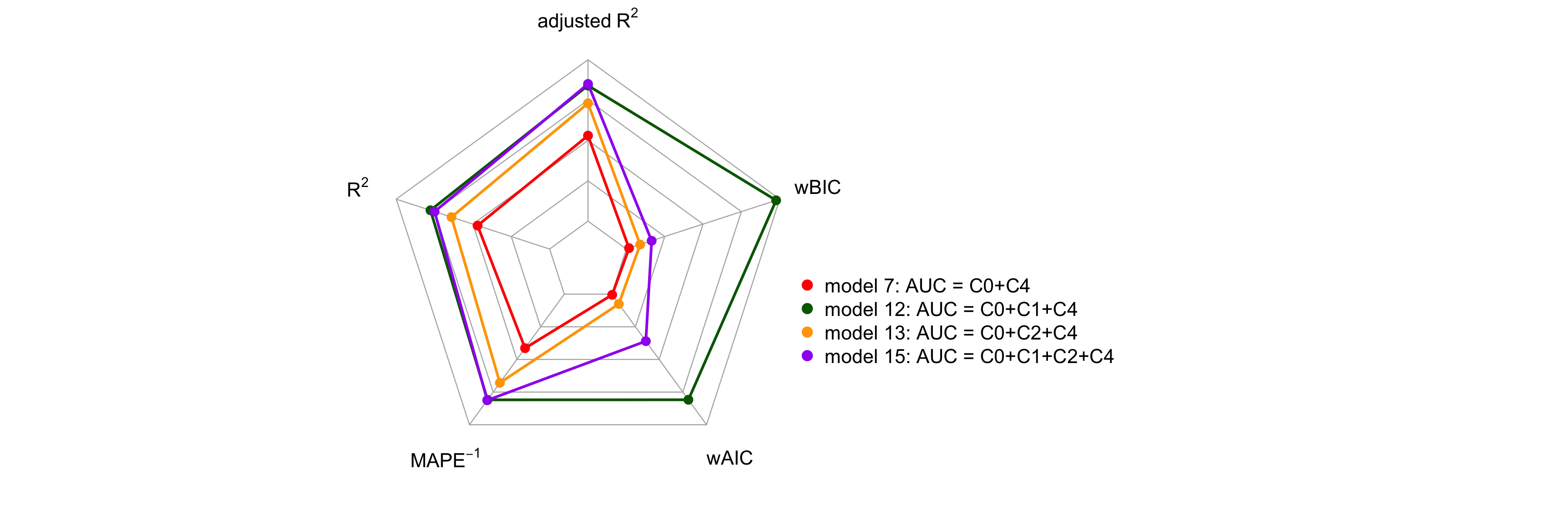

Supplement: Supplementary file 1 [file Image1.TIFF]

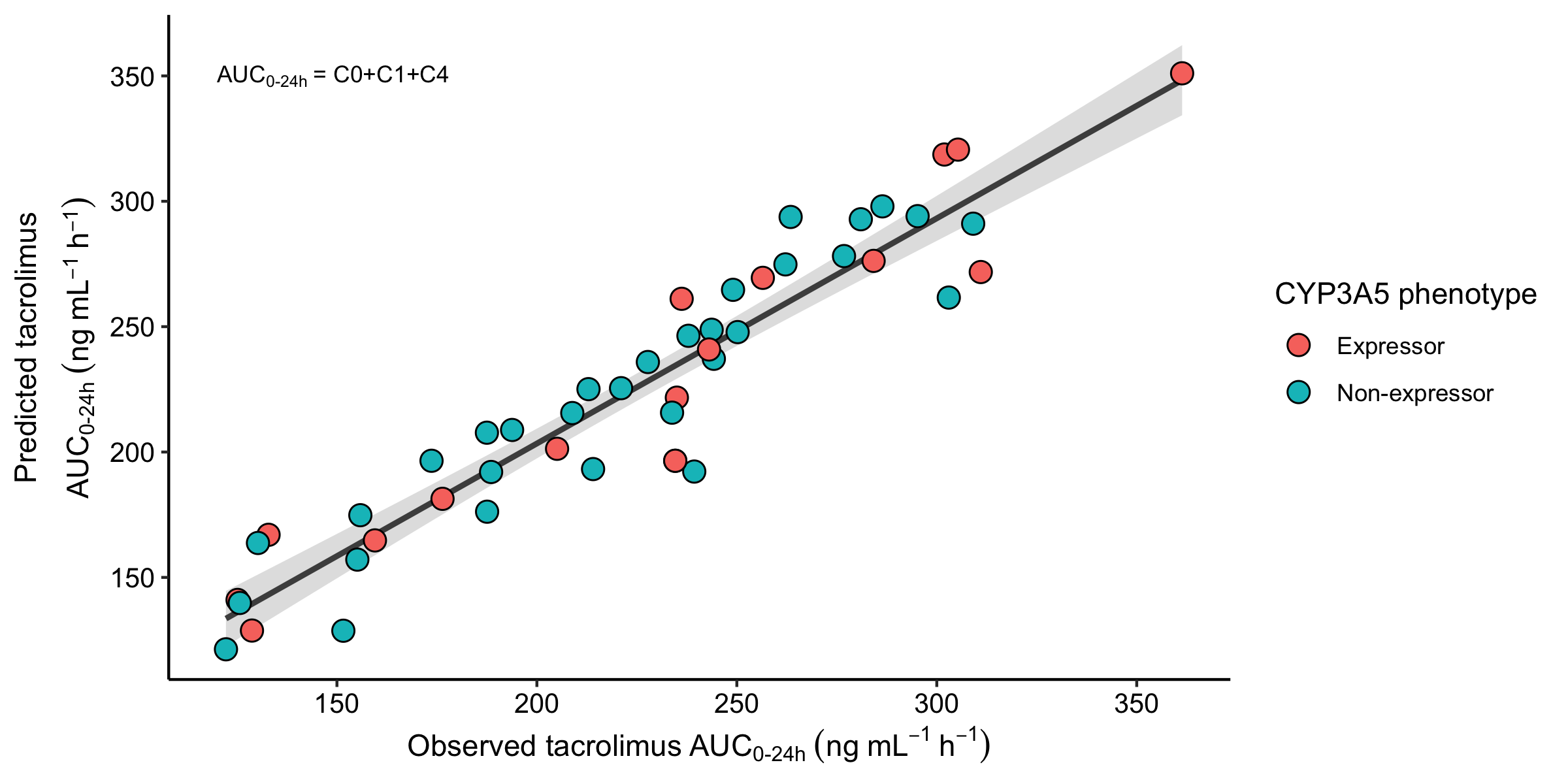

Supplement: Supplementary file 2 [file Image2.TIFF]
